# Comparative proteomic analysis of Tibetan pig spermatozoa at high and low altitudes

**DOI:** 10.1186/s12864-019-5873-0

**Published:** 2019-07-10

**Authors:** Yanling Zhao, Xiaoli Lu, Zhipeng Cheng, Mengfang Tian, Yangzong Qiangba Qiang, Qiang Fu, Zili Ren

**Affiliations:** 1 College of Animal Science, Tibet Agriculture and Animal Husbandry University, Linzhi, Tibet 860000 People’s Republic of China; 2 State Key Laboratory of Subtropical Agro-Bioresource Conservation and Utilization, Guangxi University, Nanning, Guangxi Province 530004 People’s Republic of China

**Keywords:** Tibetan pig, spermatozoa, comparative proteomics, TMT, high-altitude adaptability

## Abstract

**Background:**

To illuminate the mechanisms underlying the high-altitude tolerance of Tibetan pig spermatozoa, proteomes of spermatozoa from Tibetan pigs raised in high and low altitudes were compared using a tandem mass tag (TMT)-labeled quantitative proteomics approach.

**Results:**

A total of 77 differentially expressed proteins (DEPs) were identified. Gene Ontology (GO) analysis revealed DEPs that were predominantly associated with the actin cytoskeleton, the tricarboxylic acid (TCA) cycle, and adenosine triphosphate (ATP) metabolism, and were from 12 enriched Kyoto Encyclopedia of Genes and Genomes (KEGG) pathways. Three subnetworks were significantly enriched and 10 centric proteins were identified by protein-protein interaction (PPI) network analysis. Relative expression levels of the proteins (ATP5H, CYCS, MYH9 and FN1) were confirmed using Western blotting.

**Conclusions:**

Our study is the first to use a tandem mass tag (TMT) approach to analyze Tibetan pig spermatozoa, and provides a foundation to understand the mechanisms underlying the reproductive adaptations of Tibetan pigs to high-altitude environments.

**Electronic supplementary material:**

The online version of this article (10.1186/s12864-019-5873-0) contains supplementary material, which is available to authorized users.

## Background

The Qinghai-Tibetan plateau is an extreme environment with an average altitude of more than 4000 m [1]. High-altitude conditions with low air pressure, a reduced oxygen level, intense sun radiation, bad weather, etc., are key factors that affect the survival of domestic animals. Tibetan pigs are a unique and geographically isolated pig breed that inhabits high-altitude regions of the Qinghai-Tibetan plateau. They exhibit heritable adaptations to their high-altitude environments as a result of natural selection [2]. A series of studies have identified distinct physiological traits that contribute to the survival of Tibetan pigs on a high plateau [3, 4]. In addition, transcriptional and proteomic analyses have revealed the expression of hypoxia regulators to enable adaptation to high-altitude conditions [5–8].

With respect to mammalian reproduction, hypoxic stress affects testicular function by triggering several compensatory systems such as angiogenesis and reactive oxygen species (ROS). In addition, susceptibility to low oxygen levels is thought to cause focal damage to the seminiferous tubules [9]. However, how Tibetan pig spermatozoa have adapted to the high-altitude environment has yet to be investigated. Proteomics technology is a powerful tool to investigate protein variations on a large scale. In this study, we utilized multidimensional protein identification technology (MudPIT) combined with a tandem mass tag (TMT) approach to identify differentially expressed proteins (DEPs) in sperm from Tibetan pigs raised in high and low altitude areas. We hope that our findings will further the understanding of the effects of the high-altitude environment on sperm physiology and will help explain the high-altitude reproductive adaptations of Tibetan pigs.

## Results

### Comparison of semen quality in HT and LT pigs

Semen samples from high-altitude plateau area (HT, Linzhi city, 3000 m) and a low-altitude plain area (LT, moved from a high altitude to a low altitude for more than 5 generations, Beijing city, 100 m) Tibetan pigs were analyzed using a CASA system. No significant differences in sperm concentration, motility or abnormalities were observed between the HT and LT pigs, although the average path velocity (VAP, μm/s) of sperm from the HT pigs was statistically lower than that of the sperm from the LT pigs (*P* < 0.05) (Table 1). These results indicate that the male reproductive system of Tibetan pigs was adapted to high-altitude conditions.

**Table 1:** Semen analysis from HT and LT pigs

Semen	Sperm concentration(10^8^/mL)	Sperm motility	Sperm VAP (μm/s)	Sperm abnormality(%)
HT pigs	3.82 ± 2.10a	0.8212 ± 0.1250a	15.8 ± 2.14b	8.86 ± 6.64a
LT pigs	3.92 ± 1.87a	0.8426 ± 0.1321a	21.8 ± 4.90a	8.19 ± 7.59a

*Note: Between the HT pigs (*n* = 10) and the LT pigs (*n* = 10), different letters in the same column mean a significant difference (*P* < 0.05) and the same letters in the same column means no significant difference (*P* > 0.05)

**Table 2:** Details of the 10 centric proteins in the PPI network

Protein name	UniProt Accession No.	Symbol	MW [kDa]	Calc. pI	Fold Change*	Regulation*	Functional classification
Serum albumin	P07724	ALB	69.6	6.38	0.382	Down	complement and coagulation cascades
Cytochrome c	P62897	CYCS	11.7	9.50	2.1140	UP	the actin cytoskeleton
ATP synthase subunit d	Q9DCX2	ATP5H	18.4	5.27	3.1530	UP	oxidative phosphorylation
Gelsolin	P13020	GSN	84.7	6.32	0.4911	Down	the actin cytoskeleton
NADH dehydrogenase [ubiquinone] 1 alpha subcomplex subunit 2	Q9CQ75	NDUFA2	11.1	9.91	2.0884	UP	oxidative phosphorylation
Actin-like protein 7B	Q9QY83	ACTL7B	45.5	5.59	2.2407	UP	the actin cytoskeleton
Actin-related protein T2	Q9D9L5	ACTRT2	41.7	5.85	2.0865	UP	the actin cytoskeleton
Myosin-9	Q8VDD5	MYH9	224.9	5.77	0.3635	Down	the actin cytoskeleton
Cytochrome c oxidase subunit 6C	Q9CPQ1	COX6C	8.6	10.14	2.0688	UP	oxidative phosphorylation
Fibronectin	P11276	FN1	272.1	5.63	0.3553	Down	complement and coagulation cascades

### DEPs identification

Overall, 76,744 spectra were obtained from the LC-MS/MS analysis. A total of 1392 proteins were identified from 5625 unique peptides by quantitative proteomic analysis (FDR < 1%). Among the proteins identified, 77 DEPs (fold changes of > 2.0 or < 0.5; *P* < 0.05) were detected from two experimental replications. Details of the identified DEPs, including the protein names and UniProt accession numbers, are listed (Additional file 1: Table S1). Among the DEPs identified, 37 proteins were upregulated and 40 proteins were downregulated in the HT pigs compared with the control group of LT pigs.

### GO annotation and KEGG enrichment of the DEFs

**Fig. 1:**
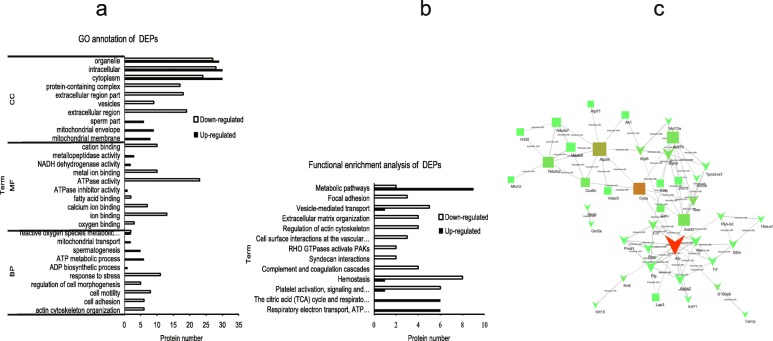
Bioinformatic analyses of differently expressed proteins (DEPs). **a** Gene ontology (GO) analysis of DEPs. **b** Functional enrichment analysis of DEPs. **c** Protein–protein interaction (PPI) network of DEPs from HT and LT pigs. The nodes represent DEPs, and the edges between the nodes indicate interactions between two connecting DEPs. The node colors indicate the betweenness of interaction the nodes: the color is redder, the betweenness is bigger, which means the influence is greater in the network. The node sizes indicate the degree of interaction between the nodes: the size is bigger, the degree is bigger, which means the stability is stronger in the network. The node shapes represent upregulated proteins (rectangle) or downregulated proteins (v). The degrees of edge thickness represent the protein–protein interaction scores

The DEFs (upregulated and downregulated) were classified by gene ontology annotation based on three categories: biological process (BP), molecular function(MF), and cellular component (CC) (Additional file 2: Table S2). Of the GO BP terms, actin cytoskeleton reorganization, protein complex involved in cell adhesion, cell motility, regulation of cell morphogenesis, and response to stress were enriched for the downregulated DEPs, while the ADP biosynthetic process, reactive oxygen species metabolic process and spermatogenesis were enriched for the upregulated DEPs. Of the MF terms, ATPase activity, oxygen binding, ion binding, calcium ion binding, and cation binding were enriched for the downregulated DEPs, while NADH dehydrogenase activity and metallopeptidase activity were enriched for the upregulated DEPs. Of the CC terms, the mitochondrial association was much more prominent for the upregulated DEPs compared with the downregulated DEPs. The extracellular region and vesicles were enriched cellular component terms for the downregulated DEPs (Fig. 1a).

To further investigate if the DEP-associated biological processes were involved in the hypoxic tolerance of sperm at high altitudes, a functional enrichment analysis was performed using the KEGG, PANTHER and Reactome databases. All 77 identified DEPs were annotated (Additional file 3: Table S3). We found that the majority of the DEPs were significantly enriched in five functional groups: the tricarboxylic acid (TCA) cycle and respiratory electron transport (CYCS, NDUA7, NDUA2, ATP5H, NDUS6, NDUFS4); plate activation, signaling and aggregation (FINC, CALU, TRFE, ALBU, PROS, APOA1, PLMN); complement and coagulation cascades (PROS, PROC, VTNC, PLMN); vesicle-mediated transport (HBB1, MYH9, TRFE, APOA1, DCTN1, ALBU); and the actin cytoskeleton (GELS, FINC, MYL12A, MYH9) (Fig. 1b).

### Protein–protein interaction (PPI) network construction and analysis

To illuminate the mechanisms of DEPs in hypoxic tolerance, we constructed a protein–protein interaction (PPI) network using STRING and Cytoscape 3.2 software (Additional file 4: Table S4). The PPI network consisted of 41 nodes and 81 edges (Fig. 1c). Ten nodes, ALB, CYCS, ATP5H, GSN, NDUFA2, ACTL7B, ACTRT2, MYH9, COX6C and FN1, were identified as centric proteins in the PPI network (Table 2). We then used the ClusterONE algorithm to detect overlapping subnetworks within the PPI network. Functional analysis revealed that proteins in three subnetworks were significantly enriched. These subnetworks were associated with complement and coagulation cascades, the actin cytoskeleton and oxidative phosphorylation (Additional file 5: Figure S1).

A number of mitochondrial proteins were found to be upregulated in the HT pigs: ATP synthase subunit d (ATP5H), adenylate kinase isoenzyme 1 (AK1), NADH dehydrogenase (NDUFS6, NDUFA7, NDUFA2), cytochrome c oxidase subunit 6C (COX6C), cytochrome c (CYCS) and mitochondrial carrier homolog 2 (MTCH2). In addition, cytoskeletal proteins localized in the sperm flagella exhibited a higher expression profile in HT pigs: actin-related proteins (ACTL7B, ACTRT2). In contrast, the expression levels of serum albumin (ALB), gelsolin (GSN), plasminogen (PLG), fibronectin (FN1) and myosin-9 (MYH9) were downregulated in HT pigs.

### Western blot validation

**Fig. 2:**
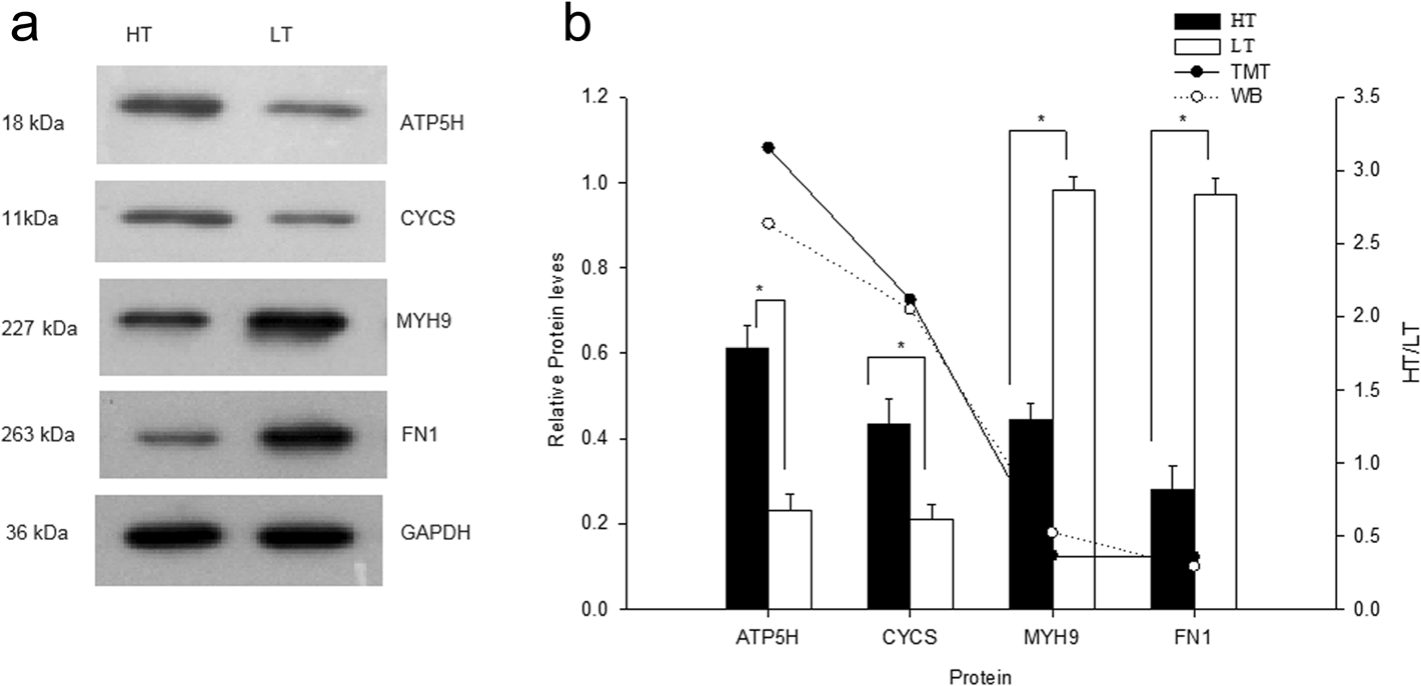
Western blot validation. **a** Western blot (WB) analysis of four proteins (ATP5H, CYCS, MYH9 and FN1). GAPDH was used as a loading control. **b** Statistical results of WB and quantitative comparisonon of TMT and WB on the four proteins. The bar chart shows statistical results of WB. The line chart represents quantitative comparisonon of TMT and WB on the four proteins. The data on WB expression levels are represented as means ± standard deviation (SD); *P < 0.05

Western blot analyses were performed to compare the expression levels of the ATP5H, CYCS, MYH9 and FN1 proteins in HT and LT pigs. The results of Western blot analysis of the four proteins are presented in Fig. 2a. The gray value of each lane of the Western blot was analyzed by using ImageJ software and the results were consistent with the analyses of the TMT quantification levels (Fig. 2b).

## Discussion

Spermatozoa are cells that are particularly susceptible to oxidative stress given their limited content of antioxidants, and hypoxia exposure has been shown to produce DNA damage in sperm cells [10]. Chronic hypobaric hypoxia (CHH) attenuates 8-oxoguanine glycosylase (OGG1) and OGG1-2a in rat spermatozoa, which may contribute to DNA damage under hypoxia exposure [11]. Hypobaric hypoxia reduces the quality of sperm DNA, including fragmentation and unpacking, and its DNA stability is diminished in the mouse [12]. Tibetan pigs have survived at high altitudes for thousands of years and have adapted to tolerate the high altitude environment. However, the molecular mechanisms underlying the adaptation of their reproductive systems to high altitude environments have not been studied in detail.

In this study, we initially compared the physical parameters of spermatozoa from high- and low-altitude Tibetan pigs. CASA analyses indicated that although the sperm VAP was significantly lower in HT pigs compared with LT pigs, their sperm counts, motility and abnormality were equivalent. These results suggested that the male reproductive system of Tibetan pigs was adapted to high-altitude conditions. To determine if there was a variation in Tibetan pig sperm protein expression levels at different altitudes, we identified and analyzed 77 differentially expressed proteins (DEPs) in sperm from Tibetan pigs living at high and low altitudes.

Previous studies have demonstrated that proteins related to sperm motility are involved in cell signaling, the regulation of cytoskeletal dynamics, and energy metabolism [13, 14]. Ras-related protein Rab-2, phospholipid hydroperoxide glutathione peroxidase (PHGPx) and mitochondrial pyruvate dehydrogenase E1 component subunit beta (PDHB) are enriched before-capacitation, and NADH dehydrogenase 1 beta subcomplex 6, mitochondrial peroxiredoxin-5 (PRDX5), mitochondrial succinyl-CoAligase [ADP-forming] subunit beta (SUCLA2), acrosin-binding protein, ropporin-1A, and spermadhesin AWN are enriched after-capacitation [15]. Essential enzymes in glycolysis/gluconeogenesis, such as HK1, ALDH2, LDHA and LDHC, are markedly upregulated in Meishan spermatozoa compared to Duroc spermatozoa [16]. Pathways associated with oxidative phosphorylation, the citrate cycle, and extracellular matrix-receptor interactions were significantly enriched [17]. Pipelines can identify putative CNV markers of fertility, especially in cases of subfertile boars [18].

In this study, we observed that the expression of several enzymes involved in energy metabolism and respiration were upregulated in HT Tibetan pig sperm compared with the sperm from the same breed of pigs raised at lower altitudes. The respiratory chain in the mitochondria is the site of oxidative phosphorylation. Here, NADH generated by the TCA cycle is oxidized to produce ATP [19]. We found that ten proteins related to the mitochondrial respiratory chain exhibited significantly higher expression levels in high-altitude Tibetan pig sperm compared with sperm from Tibetan pigs living at low altitudes. Three of these proteins (NDUFS6, NDUA2, NDUA7B) are accessory subunits of the mitochondrial NADH-coenzyme Q oxidoreductase (Complex I), and two (COX6C, CYCS) are accessory subunits of cytochrome C oxidase, the terminal enzyme of the mitochondrial respiratory chain (Complex IV). In addition, we observed a two-fold increase of ATP synthase expression (ATP5H) in HT pig sperm compared with LT pig sperm. Enhanced ATP synthase expression may serve to meet the increased energy demand required to maintain the vigorous motility of sperm in the hypoxic conditions of the high-altitude plateau area. In contrast, equivalent expression levels of glycolytic pathway enzymes were observed in HT and LT pig sperm samples. This suggests that glycolysis may not play a key role in providing the extra ATP required for maintaining sperm motility under hypoxic conditions.

Our proteomic analysis indicated that tektin-2 (TEKT2) was also found to be more abundant in HT pig sperm compared with sperm from LT pigs. Tektin-2 has been demonstrated to be vital for correct sperm flagellar structure and function and contributes to the stability and structural complexity of microtubules [20]. *Tekt2*-knockout sperm display flagellar bending and reduced motility, owing to a disruption in the dynein inner arm. Subcellular localization studies indicated that tektin-2 is associated with the surface of outer dense fibers (ODFs), rather than with axonemal tubulins [21]. Upregulation of tektin-2 expression in HT pig sperm suggests that it is required to maintain flagellum stability under hypoxic conditions. Other actin-related proteins, such as ACTL7B and ACTRT2, also showed upregulation in HT pig sperm.

We hypothesized that the upregulation of tectin-2 and actin-related proteins in Tibetan pig sperm could be a compensatory mechanism to counteract hypoxic injury. In contrast, the expression of GSN was downregulated in the sperm of HT pigs compared with LT pigs. GSN plays a key role in the rearrangement of the actin cytoskeleton during a variety of cellular processes, including cell motility [22, 23]. GSN controls actin organization by severing F-actin, capping filaments and nucleating actin assembly. It is certainly possible that the observed lower VAP of sperm from HT pigs is a direct result of lower gelsolin expression.

We also identified DEPs that are associated with posttranslational modifications. We found that the expression of proteins involved in histone methylation (DYDC1 and DPY30) and ubiquitination modification (HUWE1) were upregulated in HT pig sperm proteins. The upregulation of DYDC1 and DPY30 proteins in HT pigs suggests the Tibetan pig sperm proteins are methylated differently in a hypoxic environment. HUWE1, a ubiquitin ligase, is expressed in sperm nuclei [24], and has been shown to be a histone-binding protein. HUWE1 exhibits histone ubiquitination activity in vitro and is thought to perform a DNA modulation role during spermatogenesis [25, 26]. Previous studies have indicated that oxidative stress and DNA damage induces *Huwe1* gene expression [27–29]. We speculated that low oxygen concentrations induce HUWE1 expression to regulate DNA repair and ensure normal spermatogenesis in HT Tibetan pig sperm. Further analyses are required to investigate the detailed mechanism of how hypoxic stress regulates HUWE1 expression.

## Conclusions

**Fig. 3:**
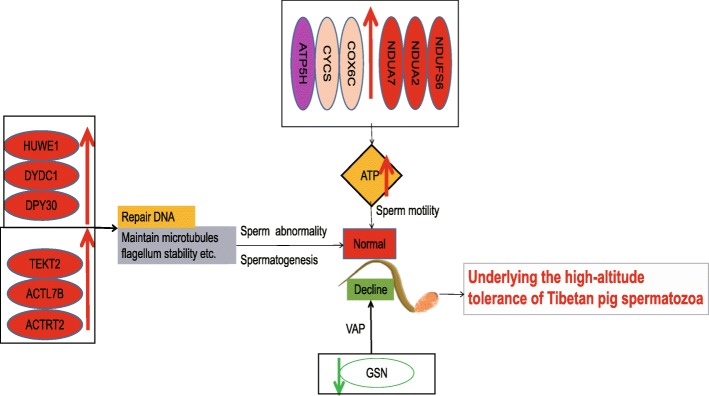
A model to present the putative mechanisms underlying the high-altitude tolerance of Tibetan pig spermatozoa. Ovals represent proteins, red arrows indicate proteins are upregulated, green arrows indicate proteins are downregulated

Hypoxic stress has no effect on the sperm counts or sperm motility of Tibetan pigs and only reduces the sperm VAP. Our comparative proteomic analyses of sperm from Tibetan pigs living in high and low altitudes have identified a number of hypoxia-sensitive proteins associated with the actin cytoskeleton, the TCA cycle, and ATP metabolism that may play key roles in the hypoxic adaptation of Tibetan pig sperm. That is, under high-altitude conditions, NDUFS6, NDUA2, NDUA7, COX6C, CYCS and ATP5H were upregulated in HT pig sperm, and enhanced ATP synthase expression may serve to meet the increased energy demand required to maintain the vigorous motility of sperm; upregulated TEKT2 can correct the sperm flagellar structure and function and contributes to the stability and structural complexity of microtubule flagellum stability, and the upregulated actin-related proteins (e.g., ACTL7B and ACTRT2) could be a compensation mechanism to counteract hypoxic injury; the upregulated HUWE1 regulates DNA repair and ensures normal spermatogenesis, while in contrast, downregulated GSN directly causes lower VAP of sperm (Fig. 3). This study provides new insights into how the reproductive mechanisms of Tibetan pigs have adapted to tolerate high-altitude environments. Further studies of the identified DEPs and their associated pathways are required to confirm and understand their roles in regulating sperm function in high altitude environments.

## Methods

All of the methods used in this study comply with the standards of the institutional guideline for ethics in animal experimentation (Rule number 86/609/EEC-24/11/86), and all experimental procedures were approved by the Institutional Animal Care and Use Committee of Tibet Agriculture & Animal Husbandry University. The institutional certification number is 12540000MB0P013721.

### Semen analysis and sperm preparation

Ten fresh semen samples, one per animal were respectively obtained from 10 Tibetan pigs (1.5 years old, approximately 35 kg, normal reproductive capacity, maintaining nutrition levels as close as possible, grazing free foraging and artificial feeding at fixed points) living in their native high-altitude plateau area (HT, Linzhi city, 3000 m) and a low-altitude plain area (LT, moved from a high altitude to a low altitude for more than 5 generations, Beijing city, 100 m) by the hand holding method. After semen collection, there was no adverse effect noted on the health and growth of the Tibetan pigs. LT and HT pigs used were raised in the Beijing Shunyi Pig Breeding Farm and the Tibet Agricultural & Animal Husbandry College Farm, respectively.

Each semen sample was separated into two portions, one for semen quality inspection and one for protein extraction. Sperm quality analyses of the HT and LT samples were performed using a Computer Assisted Semen Analysis (CASA) system (Hamilton Thorne Biosciences, MA) according to the manufacturer’s instructions. CASA was used to measure the sperm concentration, motility, and average path velocity (VAP), as well as any abnormalities.

For protein extraction, semen samples were respectively washed at 500×g for 20 min with a discontinuous (70% [v/v] and 35% [v/v]) Percoll gradient (Sigma, St Louis, MO, USA), then the sperm pellets were washed 3 times with cold phosphate-buffered-saline (PBS), to remove the seminal plasma and contamination (e.g., extender components and somatic cells such as leukocytes and testicular cells). The sperm samples (3 × 10^8^ spermatozoa per sample) were resuspended in lysis buffer (8 M urea, 4% CHAPS, 50 mM DTT and protease inhibitors, pH 8.0) at 4 °C. The lysates were sonicated, centrifuged at 10,000 *g* for 30 min to remove the insoluble material, and the supernatants were collected for further analysis. Protein concentrations were determined using a 2D Quant kit (GE Healthcare, CT, USA).

### Protein labeling and LC-MS/MS

The protein samples from the 5 HT Tibetan pig spermatozoa of HT were pooled in equal parts as a biological sample, and the same procedure was conducted for the 5 LT samples, then equal aliquots of the proteins (100 μg each) were digested using Filter-Assisted Sample Preparation (FASP) as previously described [30]. The resulting LT and HT peptides were subsequently labeled with TMT-129 and TMT-130 according to the manufacturer’s instructions (TMT 6-plex Label Reagents Kit; Thermo Fisher Scientific, Rockford, IL, USA, Cat. No.90064).

The labeled samples were then loaded onto a high-pH reversed-phase liquid chromatography (RPLC) XBridge C18 column (Waters, Milford, MA, USA) connected to a liquid chromatography system (e2695, Waters, Milford, MA, USA). The column was eluted with a 60 min gradient of 0~5% buffer B (98% acetonitrile, pH 10.0) for 5 min, 5 ~ 35% buffer B for 45 min, and 35 ~ 50% buffer B for 10 min at a flow rate of 0.7 mL/min. The fractionated peptides were desalted using Zip-Tip C18 Tips (Millipore, USA; Cat. No. 87782), suspended in buffer A (2% acetonitrile, 0.1% formic acid) and, analyzed by LC-MS/MS using a nano-LC (Easy nLC 1000, Thermo Fisher Scientific, Odense, Demark, 1.8 kV voltage) in tandem with an LTQ-Orbitrap Elite mass spectrometer (Thermo Fisher Scientific, Bremen, Germany). Survey scans in the range 150–1800 m/z were acquired with an MS resolution of 60,000 (at m/z 400) that was followed by 10 intensive precursor MS/MS scans by collision-induced dissociation (CID) fragmentation at a normalized collision energy of 35% at 30,000 resolution. The automatic gain control (AGC) target setting was 1e6 for full-MS. The second AGC target setting was 1e5 for DDA MS/MS. Moreover, dynamic exclusion was enabled for the maximum list size of 500 for duration time of a 30 s with two exclusion counts.

### Database search and bioinformatics

Since there is no Tibetan pig protein database, the MS/MS data were searched against the *Sus scrofa* UniProt database (40,710 sequences) using Proteome Discoverer™ 1.3 software. The search parameters were specified as follows: one missed enzymatic cleavage site was allowed, mass tolerance was set at 20 ppm for precursor ions and ± 0.3 Da for fragment ions, carbamidomethylation was set as a fixed modification, and oxidation and TMT-6plex were set as variable modifications. A false positive detection rate (FDR) was calculated using a decoy database search. Proteins that showed greater than a 2-fold change or less than a 0.5-fold change and a *p*-value < 0.05 were identified as differentially expressed proteins (DEPs). The DEPs data were bioinformatically analyzed while the UniProt IDs of the DEPs were converted to mouse UniProt IDs since there are few studies on gene function in pigs. The protein sequences were aligned to the Gene Ontology (GO) Consortium database for GO assignment (http://geneontology.org/). Kyoto Encyclopedia of Genes and Genomes (KEGG) pathway analysis was performed using KOBAS 3.0 (http://kobas.cbi.pku.edu.cn/) online software [31]. Protein–protein interactions (PPI) were predicted by the Search Tool for the Retrieval of Interaction Genes/Proteins (STRING 10.0) database (https://string-db.org/cgi/input.pl) [32]. PPI networks were visualized and analyzed using Cytoscape 3.2.1 software [33].

### Western blot validation

Mitochondrial ATP synthase subunit d (ATP5H), cytochrome c (CYCS), myosin-9 (MYH9) and fibronectin (FN1) expression levels were determined by Western blot analysis, with glyceraldehyde-3-phosphate dehydrogenase (GAPDH) used as a loading control. The bar line charts were created by using Sigmaplot 10.0 (Systat Software, San Jose, CA, USA).

In brief, denatured sperm proteins (40 μg) from HT and LT pigs were separated by sodium dodecyl sulfate-polyacrylamide gel electrophoresis (SDS-PAGE, a 4% stacking gel and a 10% separating gel) and transferred to Polyvinylidene fluoride (PVDF) membranes using a Hoefer TE22 blotting instrument (Hoefer, Holliston, MA, USA). The membranes were blocked overnight in blocking buffer (P0071, Beyotime Ltd., Shanghai, China), and incubated with the appropriate primary antibody (1:1000, ab173006, ab220192, ab75590, ab32419 or ab9484, Abcam, Cambridge, UK) and gently shaken at room temperature for 2 h. After three washes with phosphate buffered saline containing 0.1% Tween 20 (PBST), the membranes were incubated with the appropriate secondary antibody (1:1000, A0216 or A0208, Beyotime Ltd., Shanghai, China) for 1 h. After three washes in Tris-buffered saline with Tween 20 for 30 min, the immune complexes on the membranes were visualized using BeyoECL Plus (P0018S, A0216, Beyotime Ltd., Shanghai, China), following the manufacturer’s instructions. To determine the expression levels of ATP5H, CYCS, MYH9, and FN1 relative to GAPDH, the gray value of the bands was analyzed using ImageJ 1.44 (NIH, Bethesda, MA, USA).

### Statistical analysis

All quantitative data are presented as the means ± standard deviation (SD) from three independent experiments. A *P*-value < 0.05 was considered to be statistically significant. Statistical analyses were done using IBM SPSS Statistics version 17.0 and GraphPad Prism version 5.0. One-way Analysis of Variance (ANOVA) was used to calculate the significance of the differences between the different groups.

## Additional files

Additional file 1: **Table S1.** Details of the DEPs. (XLSX 26 kb)

Additional file 2: **Table S2.** GO Analysis of DEPs. (XLS 140 kb)

Additional file 3: **Table S3.** Functional enrichment analysis of DEPs. (XLS 89 kb)

Additional file 4: **Table S4.** The PPI relationships and scores. (XLS 37 kb)

Additional file 5: **Figure S1** Sub-networks of sperm protein-protein interaction. The green line means the PPI relation derived from STRING database, the red line means the PPI relation derived from experimential confirm. (A) complement and coagulation cascades. (B) the actin cytoskeleton. (C) oxidative phosphorylation. (TIF 915 kb)

## Data Availability

All data generated or analyzed during this study are included in this published article [and its supplementary information files].

## Abbreviations

ATP: Adenosine triphosphate
BP: Biological process
CASA: Computer Assisted Semen Analysis
CC: Cellular component
DEPs: Differentially expressed proteins
FASP: Filter-Assisted Sample Preparation
GO: Gene ontology
HT: High-altitude
KEGG: Kyoto Encyclopedia of Genes and Genomes
LC-MS/MS: Liquid chromatography-tandem mass spectrometry
LT: Low-altitude
MF: Molecular function
PPI: Protein-protein interaction
PVDF: Polyvinylidene fluoride
SDS-PAGE: Sodium dodecyl sulfate-polyacrylamide gel electrophoresis
TCA: Tricarboxylic acid
TMT: Tandem mass tag
VAP: Average path velocity

## References

[1] Yang S, Zhang H, Mao H, Yan D, Lu S, Lian L, Zhao G, Yan Y, Deng W, Shi X (2011). The local origin of the Tibetan pig and additional insights into the origin of Asian pigs. PLoS One.

[2] Li M, Tian S, Jin L, Zhou G, Li Y, Zhang Y, Wang T, Yeung CK, Chen L, Ma J (2013). Genomic analyses identify distinct patterns of selection in domesticated pigs and Tibetan wild boars. Nat Genet.

[3] Beall CM (2007). Two routes to functional adaptation: Tibetan and Andean high-altitude natives. Proc Natl Acad Sci U S A.

[4] Ge RL, Kubo K, Kobayashi T, Sekiguchi M, Honda T (1998). Blunted hypoxic pulmonary vasoconstrictive response in the rodent Ochotona curzoniae (pika) at high altitude. Am J Phys.

[5] Ai H, Huang L, Ren J (2013). Genetic diversity, linkage disequilibrium and selection signatures in chinese and Western pigs revealed by genome-wide SNP markers. PLoS One.

[6] Dong K, Yao N, Pu Y, He X, Zhao Q, Luan Y, Guan W, Rao S, Ma Y (2014). Genomic scan reveals loci under altitude adaptation in Tibetan and Dahe pigs. PLoS One.

[7] Jia C, Kong X, Koltes JE, Gou X, Yang S, Yan D, Lu S, Wei Z (2016). Gene co-expression network analysis unraveling transcriptional regulation of high-altitude adaptation of Tibetan pig. PLoS One.

[8] Zhang B, Qiangba Y, Shang P, Wang Z, Ma J, Wang L, Zhang H (2015). A comprehensive MicroRNA expression profile related to hypoxia adaptation in the Tibetan pig. PLoS One.

[9] Guan Y, Zheng X, Yang Z, Li S (2008). Change and significance of the expression of c-kit and SCF following recovery from unilateral testicular torsion in rats. Clin Invest Med.

[10] Aitken RJ, Curry BJ (2011). Redox regulation of human sperm function: from the physiological control of sperm capacitation to the etiology of infertility and DNA damage in the germ line. Antioxid Redox Signal.

[11] Farias J. G., Zepeda A., Castillo R., Figueroa E., Ademoyero O. T., Pulgar V. M. (2017). Chronic hypobaric hypoxia diminishes the expression of base excision repair OGG1 enzymes in spermatozoa. Andrologia.

[12] Vargas A, Bustos-Obregon E, Hartley R (2011). Effects of hypoxia on epididymal sperm parameters and protective role of ibuprofen and melatonin. Biol Res.

[13] Takei GL, Miyashiro D, Mukai C, Okuno M (2014). Glycolysis plays an important role in energy transfer from the base to the distal end of the flagellum in mouse sperm. J Exp Biol.

[14] Turner RM (2006). Moving to the beat: a review of mammalian sperm motility regulation. Reprod Fertil Dev.

[15] Kwon WS, Rahman MS, Lee JS, Kim J, Yoon SJ, Park YJ, You YA, Hwang S, Pang MG (2014). A comprehensive proteomic approach to identifying capacitation related proteins in boar spermatozoa. BMC Genomics.

[16] Xinhong L, Zhen L, Fu J, Wang L, Yang Q, Li P, Li Y (2018). Quantitative proteomic profiling indicates the difference in reproductive efficiency between Meishan and Duroc boar spermatozoa. Theriogenology.

[17] Feugang JM, Liao SF, Willard ST, Ryan PL (2018). In-depth proteomic analysis of boar spermatozoa through shotgun and gel-based methods. BMC Genomics.

[18] Revay T, Quach AT, Maignel L, Sullivan B, King WA (2015). Copy number variations in high and low fertility breeding boars. BMC Genomics.

[19] Mukai C, Okuno M (2004). Glycolysis plays a major role for adenosine triphosphate supplementation in mouse sperm flagellar movement. Biol Reprod.

[20] Matsuyama T, Honda Y, Doiguchi M, Iida H (2005). Molecular cloning of a new member of TEKTIN family, Tektin4, located to the flagella of rat spermatozoa. Mol Reprod Dev.

[21] Shimasaki S, Yamamoto E, Murayama E, Kurio H, Kaneko T, Shibata Y, Inai T, Iida H (2010). Subcellular localization of Tektin2 in rat sperm flagellum. Zool Sci.

[22] Kwiatkowski DJ (1999). Functions of gelsolin: motility, signaling, apoptosis, cancer. Curr Opin Cell Biol.

[23] Sun HQ, Yamamoto M, Mejillano M, Yin HL (1999). Gelsolin, a multifunctional actin regulatory protein. J Biol Chem.

[24] Chen LJ, Xu WM, Yang M, Wang K, Chen Y, Huang XJ, Ma QH (2016). HUWE1 plays important role in mouse preimplantation embryo development and the dysregulation is associated with poor embryo development in humans. Sci Rep.

[25] Liu Z, Miao D, Xia Q, Hermo L, Wing SS (2007). Regulated expression of the ubiquitin protein ligase, E3(histone)/LASU1/mule/ARF-BP1/HUWE1, during spermatogenesis. Dev Dyn.

[26] Liu Z, Oughtred R, Wing SS (2005). Characterization of E3Histone, a novel testis ubiquitin protein ligase which ubiquitinates histones. Mol Cell Biol.

[27] Kim SK, Jee BC, Kim SH (2015). Histone methylation and acetylation in ejaculated human sperm: effects of swim-up and smoking. Fertil Steril.

[28] Thompson JW, Nagel J, Hoving S, Gerrits B, Bauer A, Thomas JR, Kirschner MW, Schirle M, Luchansky SJ (2014). Quantitative Lys--Gly-Gly (diGly) proteomics coupled with inducible RNAi reveals ubiquitin-mediated proteolysis of DNA damage-inducible transcript 4 (DDIT4) by the E3 ligase HUWE1. J Biol Chem.

[29] Yi J, Lu G, Li L, Wang X, Cao L, Lin M, Zhang S, Shao G (2015). DNA damage-induced activation of CUL4B targets HUWE1 for proteasomal degradation. Nucleic Acids Res.

[30] Wisniewski JR, Zougman A, Mann M (2009). Combination of FASP and StageTip-based fractionation allows in-depth analysis of the hippocampal membrane proteome. J Proteome Res.

[31] Xie C, Mao X, Huang J, Ding Y, Wu J, Dong S, Kong L, Gao G, Li CY, Wei L (2011). KOBAS 2.0: a web server for annotation and identification of enriched pathways and diseases. Nucleic Acids Res.

[32] Szklarczyk D, Franceschini A, Kuhn M, Simonovic M, Roth A, Minguez P, Doerks T, Stark M, Muller J, Bork P (2011). The STRING database in 2011: functional interaction networks of proteins, globally integrated and scored. Nucleic Acids Res.

[33] Shannon P, Markiel A, Ozier O, Baliga NS, Wang JT, Ramage D, Amin N, Schwikowski B, Ideker T (2003). Cytoscape: a software environment for integrated models of biomolecular interaction networks. Genome Res.

