# Correction to: Comparative proteomic analysis of Tibetan pig spermatozoa at high and low altitudes

**DOI:** 10.1186/s12864-019-5993-6

**Published:** 2019-08-01

**Authors:** Yanling Zhao, Xiaoli Lu, Zhipeng Cheng, Mengfang Tian, Yangzong Qiangba, Qiang Fu, Zili Ren

**Affiliations:** 1 College of Animal Science, Tibet Agriculture and Animal Husbandry University, Linzhi, Tibet 860000 People’s Republic of China; 2 State Key Laboratory of Subtropical Agro-Bioresource Conservation and Utilization, Guangxi University, Nanning, Guangxi Province 530004 People’s Republic of China

**Correction to: Zhao et al. BMC Genomics (2019) 20:569**

**https://doi.org/10.1186/s12864-019-5873-0**

Following publication of the original article [1], the authors reported an error in one of the authors’ names. In this Correction the incorrect and correct author name are shown. The original publication of this article has been corrected.

Originally the author name was published as:

- Yangzong Qiangba Qiang

The correct author name is:

- Yangzong Qiangba
