# Supplementary figures and images for: Comparative proteomic analysis of Tibetan pig spermatozoa at high and low altitudes
Source: BMC Genomics. 2019 Jul 10;20:569. doi: 10.1186/s12864-019-5873-0 (PMC6617692; doi:10.1186/s12864-019-5873-0)

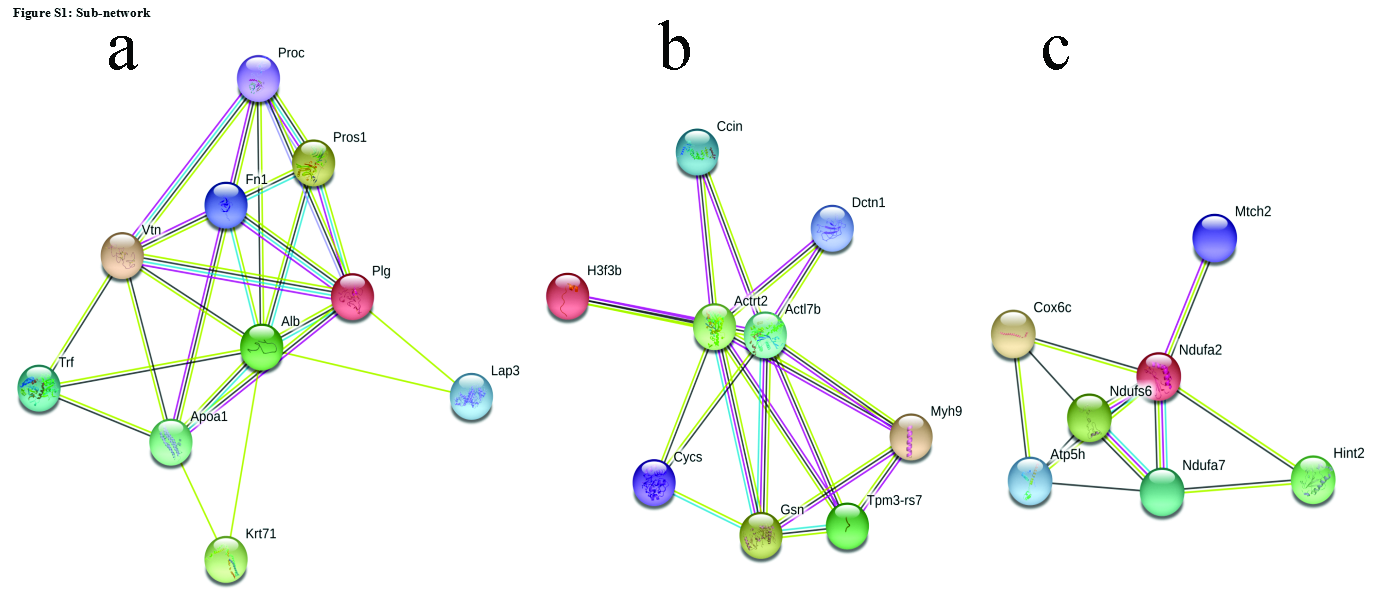

Supplement: Supplementary file 5 — Figure S1 Sub-networks of sperm protein-protein interaction. The green line means the PPI relation derived from STRING database, the red line means the PPI relation derived from experimential confirm. (A) complement and coagulation cascades. (B) the actin cytoskeleton. (C) oxidative phosphorylation. (TIF 915 kb) [file 12864_2019_5873_MOESM5_ESM.tif]
